# Supplementary material for: Resolving the full spectrum of human genome variation using Linked-Reads
Source: Genome Res. 2019 Apr;29(4):635–45. doi: 10.1101/gr.234443.118 (PMC6442396; doi:10.1101/gr.234443.118)
Supplement: Supplemental Material [file supp_gr.234443.118_Supplemental_longranger-2.2.zip › longranger-2.2.tar.bz2!/lr2.2-public/lib/stan/cmdstan/stan/doc/api/html/agrad_2rev_2matrix_2mdivide__left__tri_8hpp_source.html]

Stan: src/stan/agrad/rev/matrix/mdivide\_left\_tri.hpp Source File


|  |  |
| --- | --- |
|  | Stan  2.5.0  probability, sampling & optimization |


- Main Page
- Related Pages
- Namespaces
- Classes
- Files

- File List
- File Members

All Classes Namespaces Files Functions Variables Typedefs Enumerations Enumerator Friends Macros Pages

- src
- stan
- agrad
- rev
- matrix

mdivide\_left\_tri.hpp

Go to the documentation of this file.

1 #ifndef STAN\_\_AGRAD\_\_REV\_\_MATRIX\_\_MDIVIDE\_LEFT\_TRI\_HPP

2 #define STAN\_\_AGRAD\_\_REV\_\_MATRIX\_\_MDIVIDE\_LEFT\_TRI\_HPP

3

4 #include <vector>

5 #include <stan/math/matrix/Eigen.hpp>

6 #include <stan/math/matrix/typedefs.hpp>

7 #include <stan/math/error\_handling/matrix/check\_multiplicable.hpp>

8 #include <stan/math/error\_handling/matrix/check\_square.hpp>

9 #include <stan/agrad/rev/var.hpp>

10 #include <stan/agrad/rev/matrix/typedefs.hpp>

11

12 namespace stan {

13  namespace agrad {

14

15  namespace {

16  template <int TriView,int R1,int C1,int R2,int C2>

17  class mdivide\_left\_tri\_vv\_vari : public vari {

18  public:

19  int M\_; // A.rows() = A.cols() = B.rows()

20  int N\_; // B.cols()

21  double\* A\_;

22  double\* C\_;

23  vari\*\* \_variRefA;

24  vari\*\* \_variRefB;

25  vari\*\* \_variRefC;

26

27  mdivide\_left\_tri\_vv\_vari(const Eigen::Matrix<var,R1,C1> &A,

28  const Eigen::Matrix<var,R2,C2> &B)

29  : vari(0.0),

30  M\_(A.rows()),

31  N\_(B.cols()),

32  A\_((double\*)stan::agrad::memalloc\_.alloc(sizeof(double)

33  \* A.rows() \* A.cols())),

34  C\_((double\*)stan::agrad::memalloc\_.alloc(sizeof(double)

35  \* B.rows() \* B.cols())),

36  \_variRefA((vari\*\*)stan::agrad::memalloc\_.alloc(sizeof(vari\*)

37  \* A.rows()

38  \* (A.rows() + 1) / 2)),

39  \_variRefB((vari\*\*)stan::agrad::memalloc\_.alloc(sizeof(vari\*)

40  \* B.rows() \* B.cols())),

41  \_variRefC((vari\*\*)stan::agrad::memalloc\_.alloc(sizeof(vari\*)

42  \* B.rows() \* B.cols()))

43  {

44  using Eigen::Matrix;

45  using Eigen::Map;

46

47  size\_t pos = 0;

48  if (TriView == Eigen::Lower) {

49  for (size\_type j = 0; j < M\_; j++)

50  for (size\_type i = j; i < M\_; i++)

51  \_variRefA[pos++] = A(i,j).vi\_;

52  } else if (TriView == Eigen::Upper) {

53  for (size\_type j = 0; j < M\_; j++)

54  for (size\_type i = 0; i < j+1; i++)

55  \_variRefA[pos++] = A(i,j).vi\_;

56  }

57

58  pos = 0;

59  for (size\_type j = 0; j < M\_; j++) {

60  for (size\_type i = 0; i < M\_; i++) {

61  A\_[pos++] = A(i,j).val();

62  }

63  }

64

65  pos = 0;

66  for (size\_type j = 0; j < N\_; j++) {

67  for (size\_type i = 0; i < M\_; i++) {

68  \_variRefB[pos] = B(i,j).vi\_;

69  C\_[pos++] = B(i,j).val();

70  }

71  }

72

73  Matrix<double,R1,C2> C(M\_,N\_);

74  C = Map<Matrix<double,R1,C2> >(C\_,M\_,N\_);

75

76  C = Map<Matrix<double,R1,C1> >(A\_,M\_,M\_)

77  .template triangularView<TriView>().solve(C);

78

79  pos = 0;

80  for (size\_type j = 0; j < N\_; j++) {

81  for (size\_type i = 0; i < M\_; i++) {

82  C\_[pos] = C(i,j);

83  \_variRefC[pos] = new vari(C\_[pos],false);

84  pos++;

85  }

86  }

87

88  }

89

90  virtual void chain() {

91  using Eigen::Matrix;

92  using Eigen::Map;

93  Matrix<double,R1,C1> adjA(M\_,M\_);

94  Matrix<double,R2,C2> adjB(M\_,N\_);

95  Matrix<double,R1,C2> adjC(M\_,N\_);

96

97  size\_t pos = 0;

98  for (size\_type j = 0; j < adjC.cols(); j++)

99  for (size\_type i = 0; i < adjC.rows(); i++)

100  adjC(i,j) = \_variRefC[pos++]->adj\_;

101

102  adjB = Map<Matrix<double,R1,C1> >(A\_,M\_,M\_)

103  .template triangularView<TriView>().transpose().solve(adjC);

104  adjA.noalias() = -adjB

105  \* Map<Matrix<double,R1,C2> >(C\_,M\_,N\_).transpose();

106

107  pos = 0;

108  if (TriView == Eigen::Lower) {

109  for (size\_type j = 0; j < adjA.cols(); j++)

110  for (size\_type i = j; i < adjA.rows(); i++)

111  \_variRefA[pos++]->adj\_ += adjA(i,j);

112  } else if (TriView == Eigen::Upper) {

113  for (size\_type j = 0; j < adjA.cols(); j++)

114  for (size\_type i = 0; i < j+1; i++)

115  \_variRefA[pos++]->adj\_ += adjA(i,j);

116  }

117

118  pos = 0;

119  for (size\_type j = 0; j < adjB.cols(); j++)

120  for (size\_type i = 0; i < adjB.rows(); i++)

121  \_variRefB[pos++]->adj\_ += adjB(i,j);

122  }

123  };

124

125  template <int TriView,int R1,int C1,int R2,int C2>

126  class mdivide\_left\_tri\_dv\_vari : public vari {

127  public:

128  int M\_; // A.rows() = A.cols() = B.rows()

129  int N\_; // B.cols()

130  double\* A\_;

131  double\* C\_;

132  vari\*\* \_variRefB;

133  vari\*\* \_variRefC;

134

135  mdivide\_left\_tri\_dv\_vari(const Eigen::Matrix<double,R1,C1> &A,

136  const Eigen::Matrix<var,R2,C2> &B)

137  : vari(0.0),

138  M\_(A.rows()),

139  N\_(B.cols()),

140  A\_((double\*)stan::agrad::memalloc\_.alloc(sizeof(double)

141  \* A.rows() \* A.cols())),

142  C\_((double\*)stan::agrad::memalloc\_.alloc(sizeof(double)

143  \* B.rows() \* B.cols())),

144  \_variRefB((vari\*\*)stan::agrad::memalloc\_.alloc(sizeof(vari\*)

145  \* B.rows() \* B.cols())),

146  \_variRefC((vari\*\*)stan::agrad::memalloc\_.alloc(sizeof(vari\*)

147  \* B.rows() \* B.cols()))

148  {

149  using Eigen::Matrix;

150  using Eigen::Map;

151

152  size\_t pos = 0;

153  for (size\_type j = 0; j < M\_; j++) {

154  for (size\_type i = 0; i < M\_; i++) {

155  A\_[pos++] = A(i,j);

156  }

157  }

158

159  pos = 0;

160  for (size\_type j = 0; j < N\_; j++) {

161  for (size\_type i = 0; i < M\_; i++) {

162  \_variRefB[pos] = B(i,j).vi\_;

163  C\_[pos++] = B(i,j).val();

164  }

165  }

166

167  Matrix<double,R1,C2> C(M\_,N\_);

168  C = Map<Matrix<double,R1,C2> >(C\_,M\_,N\_);

169

170  C = Map<Matrix<double,R1,C1> >(A\_,M\_,M\_)

171  .template triangularView<TriView>().solve(C);

172

173  pos = 0;

174  for (size\_type j = 0; j < N\_; j++) {

175  for (size\_type i = 0; i < M\_; i++) {

176  C\_[pos] = C(i,j);

177  \_variRefC[pos] = new vari(C\_[pos],false);

178  pos++;

179  }

180  }

181  }

182

183  virtual void chain() {

184  using Eigen::Matrix;

185  using Eigen::Map;

186  Matrix<double,R2,C2> adjB(M\_,N\_);

187  Matrix<double,R1,C2> adjC(M\_,N\_);

188

189  size\_t pos = 0;

190  for (size\_type j = 0; j < adjC.cols(); j++)

191  for (size\_type i = 0; i < adjC.rows(); i++)

192  adjC(i,j) = \_variRefC[pos++]->adj\_;

193

194  adjB = Map<Matrix<double,R1,C1> >(A\_,M\_,M\_)

195  .template triangularView<TriView>().transpose().solve(adjC);

196

197  pos = 0;

198  for (size\_type j = 0; j < adjB.cols(); j++)

199  for (size\_type i = 0; i < adjB.rows(); i++)

200  \_variRefB[pos++]->adj\_ += adjB(i,j);

201  }

202  };

203

204  template <int TriView,int R1,int C1,int R2,int C2>

205  class mdivide\_left\_tri\_vd\_vari : public vari {

206  public:

207  int M\_; // A.rows() = A.cols() = B.rows()

208  int N\_; // B.cols()

209  double\* A\_;

210  double\* C\_;

211  vari\*\* \_variRefA;

212  vari\*\* \_variRefC;

213

214  mdivide\_left\_tri\_vd\_vari(const Eigen::Matrix<var,R1,C1> &A,

215  const Eigen::Matrix<double,R2,C2> &B)

216  : vari(0.0),

217  M\_(A.rows()),

218  N\_(B.cols()),

219  A\_((double\*)stan::agrad::memalloc\_.alloc(sizeof(double)

220  \* A.rows() \* A.cols())),

221  C\_((double\*)stan::agrad::memalloc\_.alloc(sizeof(double)

222  \* B.rows() \* B.cols())),

223  \_variRefA((vari\*\*)stan::agrad::memalloc\_.alloc(sizeof(vari\*)

224  \* A.rows()

225  \* (A.rows() + 1) / 2)),

226  \_variRefC((vari\*\*)stan::agrad::memalloc\_.alloc(sizeof(vari\*)

227  \* B.rows() \* B.cols()))

228  {

229  using Eigen::Matrix;

230  using Eigen::Map;

231

232  size\_t pos = 0;

233  if (TriView == Eigen::Lower) {

234  for (size\_type j = 0; j < M\_; j++)

235  for (size\_type i = j; i < M\_; i++)

236  \_variRefA[pos++] = A(i,j).vi\_;

237  } else if (TriView == Eigen::Upper) {

238  for (size\_type j = 0; j < M\_; j++)

239  for (size\_type i = 0; i < j+1; i++)

240  \_variRefA[pos++] = A(i,j).vi\_;

241  }

242

243  pos = 0;

244  for (size\_type j = 0; j < M\_; j++) {

245  for (size\_type i = 0; i < M\_; i++) {

246  A\_[pos++] = A(i,j).val();

247  }

248  }

249

250  Matrix<double,R1,C2> C(M\_,N\_);

251  C = Map<Matrix<double,R1,C1> >(A\_,M\_,M\_)

252  .template triangularView<TriView>().solve(B);

253

254  pos = 0;

255  for (size\_type j = 0; j < N\_; j++) {

256  for (size\_type i = 0; i < M\_; i++) {

257  C\_[pos] = C(i,j);

258  \_variRefC[pos] = new vari(C\_[pos],false);

259  pos++;

260  }

261  }

262  }

263

264  virtual void chain() {

265  using Eigen::Matrix;

266  using Eigen::Map;

267  Matrix<double,R1,C1> adjA(M\_,M\_);

268  Matrix<double,R1,C2> adjC(M\_,N\_);

269

270  size\_t pos = 0;

271  for (size\_type j = 0; j < adjC.cols(); j++)

272  for (size\_type i = 0; i < adjC.rows(); i++)

273  adjC(i,j) = \_variRefC[pos++]->adj\_;

274

275  adjA.noalias() = -Map<Matrix<double,R1,C1> >(A\_,M\_,M\_)

276  .template triangularView<TriView>()

277  .transpose().solve(adjC\*Map<Matrix<double,R1,C2> >(C\_,M\_,N\_).transpose());

278

279  pos = 0;

280  if (TriView == Eigen::Lower) {

281  for (size\_type j = 0; j < adjA.cols(); j++)

282  for (size\_type i = j; i < adjA.rows(); i++)

283  \_variRefA[pos++]->adj\_ += adjA(i,j);

284  } else if (TriView == Eigen::Upper) {

285  for (size\_type j = 0; j < adjA.cols(); j++)

286  for (size\_type i = 0; i < j+1; i++)

287  \_variRefA[pos++]->adj\_ += adjA(i,j);

288  }

289  }

290  };

291  }

292

293  template <int TriView,int R1,int C1,int R2,int C2>

294  inline

295  Eigen::Matrix<var,R1,C2>

296  mdivide\_left\_tri(const Eigen::Matrix<var,R1,C1> &A,

297  const Eigen::Matrix<var,R2,C2> &b) {

298  Eigen::Matrix<var,R1,C2> res(b.rows(),b.cols());

299

300  stan::math::check\_square("mdivide\_left\_tri(%1%)",A,"A",(double\*)0);

301  stan::math::check\_multiplicable("mdivide\_left\_tri(%1%)",A,"A",

302  b,"b",(double\*)0);

303

304  // NOTE: this is not a memory leak, this vari is used in the

305  // expression graph to evaluate the adjoint, but is not needed

306  // for the returned matrix. Memory will be cleaned up with the arena allocator.

307  mdivide\_left\_tri\_vv\_vari<TriView,R1,C1,R2,C2> \*baseVari = new mdivide\_left\_tri\_vv\_vari<TriView,R1,C1,R2,C2>(A,b);

308

309  size\_t pos = 0;

310  for (size\_type j = 0; j < res.cols(); j++)

311  for (size\_type i = 0; i < res.rows(); i++)

312  res(i,j).vi\_ = baseVari->\_variRefC[pos++];

313

314  return res;

315  }

316  template <int TriView,int R1,int C1,int R2,int C2>

317  inline

318  Eigen::Matrix<var,R1,C2>

319  mdivide\_left\_tri(const Eigen::Matrix<double,R1,C1> &A,

320  const Eigen::Matrix<var,R2,C2> &b) {

321  Eigen::Matrix<var,R1,C2> res(b.rows(),b.cols());

322

323  stan::math::check\_square("mdivide\_left\_tri(%1%)",A,"A",(double\*)0);

324  stan::math::check\_multiplicable("mdivide\_left\_tri(%1%)",A,"A",

325  b,"b",(double\*)0);

326

327  // NOTE: this is not a memory leak, this vari is used in the

328  // expression graph to evaluate the adjoint, but is not needed

329  // for the returned matrix. Memory will be cleaned up with the arena allocator.

330  mdivide\_left\_tri\_dv\_vari<TriView,R1,C1,R2,C2> \*baseVari = new mdivide\_left\_tri\_dv\_vari<TriView,R1,C1,R2,C2>(A,b);

331

332  size\_t pos = 0;

333  for (size\_type j = 0; j < res.cols(); j++)

334  for (size\_type i = 0; i < res.rows(); i++)

335  res(i,j).vi\_ = baseVari->\_variRefC[pos++];

336

337  return res;

338  }

339  template <int TriView,int R1,int C1,int R2,int C2>

340  inline

341  Eigen::Matrix<var,R1,C2>

342  mdivide\_left\_tri(const Eigen::Matrix<var,R1,C1> &A,

343  const Eigen::Matrix<double,R2,C2> &b) {

344  Eigen::Matrix<var,R1,C2> res(b.rows(),b.cols());

345

346  stan::math::check\_square("mdivide\_left\_tri(%1%)",A,"A",(double\*)0);

347  stan::math::check\_multiplicable("mdivide\_left\_tri(%1%)",A,"A",

348  b,"b",(double\*)0);

349

350  // NOTE: this is not a memory leak, this vari is used in the

351  // expression graph to evaluate the adjoint, but is not needed

352  // for the returned matrix. Memory will be cleaned up with the arena allocator.

353  mdivide\_left\_tri\_vd\_vari<TriView,R1,C1,R2,C2> \*baseVari = new mdivide\_left\_tri\_vd\_vari<TriView,R1,C1,R2,C2>(A,b);

354

355  size\_t pos = 0;

356  for (size\_type j = 0; j < res.cols(); j++)

357  for (size\_type i = 0; i < res.rows(); i++)

358  res(i,j).vi\_ = baseVari->\_variRefC[pos++];

359

360  return res;

361  }

362

363  }

364 }

365 #endif

check\_multiplicable.hpp

typedefs.hpp

stan::agrad::mdivide\_left\_tri

Eigen::Matrix< var, R1, C2 > mdivide\_left\_tri(const Eigen::Matrix< var, R1, C1 > &A, const Eigen::Matrix< var, R2, C2 > &b)

**Definition:** mdivide\_left\_tri.hpp:296

typedefs.hpp

stan::agrad::memalloc\_

memory::stack\_alloc memalloc\_

**Definition:** var\_stack.cpp:16

N\_

int N\_

**Definition:** mdivide\_left\_tri.hpp:20

stan::math::check\_multiplicable

bool check\_multiplicable(const char \*function, const T1 &y1, const char \*name1, const T2 &y2, const char \*name2, T\_result \*result)

**Definition:** check\_multiplicable.hpp:15

\_variRefC

vari \*\* \_variRefC

**Definition:** mdivide\_left\_tri.hpp:25

stan::math::transpose

Eigen::Matrix< T, C, R > transpose(const Eigen::Matrix< T, R, C > &m)

**Definition:** transpose.hpp:12

M\_

int M\_

**Definition:** mdivide\_left\_tri.hpp:19

\_variRefA

vari \*\* \_variRefA

**Definition:** mdivide\_left\_tri.hpp:23

stan::agrad::size\_type

Eigen::Matrix< double, Eigen::Dynamic, Eigen::Dynamic >::Index size\_type

**Definition:** typedefs.hpp:14

stan::math::rows

size\_t rows(const Eigen::Matrix< T, R, C > &m)

**Definition:** rows.hpp:12

var.hpp

stan::math::cols

size\_t cols(const Eigen::Matrix< T, R, C > &m)

**Definition:** cols.hpp:12

C\_

double \* C\_

**Definition:** mdivide\_left\_tri.hpp:22

Eigen.hpp

\_variRefB

vari \*\* \_variRefB

**Definition:** mdivide\_left\_tri.hpp:24

A\_

double \* A\_

**Definition:** mdivide\_left\_tri.hpp:21

stan::math::check\_square

bool check\_square(const char \*function, const Eigen::Matrix< T\_y, Eigen::Dynamic, Eigen::Dynamic > &y, const char \*name, T\_result \*result)

Return true if the specified matrix is square.

**Definition:** check\_square.hpp:24

check\_square.hpp


---

[ Stan Home Page ]

*© 2011–2014,
Stan Development Team.*
